# Involvement of the Red Nucleus in the Compensation of Parkinsonism may Explain why Primates can develop Stable Parkinson’s Disease

**DOI:** 10.1038/s41598-018-37381-1

**Published:** 2019-01-29

**Authors:** Ingrid H. C. H. M. Philippens, Jacqueline A. Wubben, Sigrid K. Franke, Sam Hofman, Jan A. M. Langermans

**Affiliations:** 1 Animal Science Department, Biomedical Primate Research Centre (BPRC), P.O. Box 3306, 2280 GH Rijswijk, The Netherlands; 2 Department of Immunobiology, Biomedical Primate Research Centre (BPRC), P.O. Box 3306, 2280 GH Rijswijk, The Netherlands; 3 Department of Molecular and Cellular Neurobiology, Center for Neurogenomics and Cognitive Research, Neuroscience Campus Amsterdam, VU University, Amsterdam, The Netherlands

## Abstract

Neurological compensatory mechanisms help our brain to adjust to neurodegeneration as in Parkinson’s disease. It is suggested that the compensation of the damaged striato-thalamo-cortical circuit is focused on the intact thalamo-rubro-cerebellar pathway as seen during presymptomatic Parkinson, paradoxical movement and sensorimotor rhythm (SMR). Indeed, the size of the red nucleus, connecting the cerebellum with the cerebral cortex, is larger in Parkinson’s disease patients suggesting an increased activation of this brain area. Therefore, the red nucleus was examined in MPTP-induced parkinsonian marmoset monkeys during the presymptomatic stage and after SMR activation by neurofeedback training. We found a reverse significant correlation between the early expression of parkinsonian signs and the size of the parvocellular part of the red nucleus, which is predominantly present in human and non-human primates. In quadrupedal animals it consists mainly of the magnocellular part. Furthermore, SMR activation, that mitigated parkinsonian signs, further increased the size of the red nucleus in the marmoset monkey. This plasticity of the brain helps to compensate for dysfunctional movement control and can be a promising target for compensatory treatment with neurofeedback technology, vibrotactile stimulation or DBS in order to improve the quality of life for Parkinson’s disease patients.

## Introduction

Parkinson’s disease (PD) is a major progressive disorder affecting the central nervous system by specific degeneration of dopamine producing neurons in the *substantia nigra*, which govern the control of muscle movement. Treatments against this motor disorder are still based on increasing dopamine neurotransmission capacity in the brain by pharmacological intervention, such as Levodopa (L-DOPA) that reduces the PD symptoms. Although treatment with L-DOPA initially improves PD symptoms, long-term use frequently causes excessive, spasmodic movements (dyskinesia) and other side effects^1^. Therefore, there is an urgent need for new treatment modalities, such as targeting neurological compensation mechanism that assist our brain in adjusting to ever-changing conditions and events around us, including age-related neurodegeneration. It is known that neurodegeneration is already ongoing for years resulting in more than 60% of dopamine producing cell loss in the *substantia nigra* at the time of diagnosis^2^. This does not imply that one needs only half of the neurons of the *substantia nigra*. The reason we don’t see any motor symptoms during the pre-clinical stage may be a result of a compensation mechanism, which is called ‘presymptomatic PD’^3^. These compensation mechanism can happen on cellular, morphological or functional level. Changes at the synaptic level occurs in order to maintain homeostasis of the dopamine system. Also the hyperdirect pathway, with direct connections from the cortex to the subthalamic nucleus (STN), play a role in compensation^4^. However, compensation can also occur by recruitment of other brain regions that are not associated with the damaged brain area^3^. These mechanisms serve to maintain optimal performance of the impaired function.

Therefore, activation of compensatory mechanisms, in addition to ameliorating the dopamine depletion in PD, may represent a joint strategy for minimization of disability and improvement of the quality of life of the patient.

Brain imaging techniques have revealed that cerebellar circuits compensate for impaired basal ganglia function as seen in PD. This is explained by altered connectivity within the brain through which motor function can be maintained^3^.

A comparable mechanism is seen during paradoxical movement. PD patients who are not able to walk can start a movement by external visual guidance such as lines drawn on the floor. The pathways relaying visual stimuli can bypass the damaged basal ganglia and allow an intact cerebellar circuit to be used for visuomotor control^5^. Compensatory pathways using external cues and gait are often making use of the cortico-parieto-frontal circuitry and cerebellum^6^.

**Figure 1:**
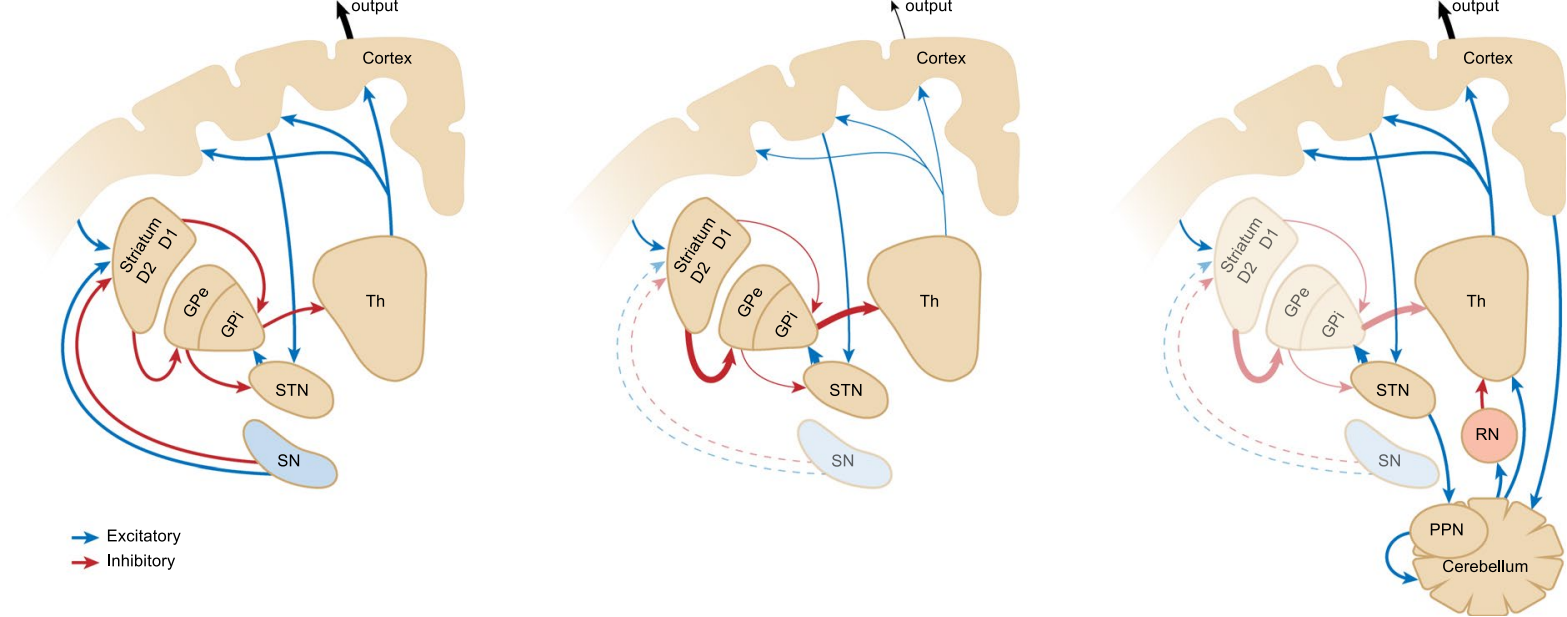
Schematic connections of striato-thalamo-cortical vs cerebello-rubro-thalamo-cortical circuitry. Left: Normal functional striato-thalamo-cortical circuit. Middle: Dysfunctional striato-thalamo-cortical circuit as seen in PD. Right: An activated compensatory cerebellar circuit for recovered motor function. Blue lines: excitatory connections; red lines: inhibitory connections. A damaged substantia nigra (SN) causes increased inhibition from the globus pallidus internal (GPi) to the thalamus (Th), resulting in less cortical innervation. Feedback loops from the cortex to the subthalamic nucleus (STN) and cerebellum via the pedunculopontine nucleus (PPN) are responsible for compensation mechanisms that overrule the damaged striato-thalamocortical circuitry via the red nucleus (RN). Reduced muscle tone decreases RN activity, resulting in thalamic hyperpolarization with reciprocal burst activity which is propagated to the sensorimotor cortex as SMR^12^.

Another mechanism to compensate for the damaged basal ganglia is stimulation of the sensorimotor rhythm (SMR)^7–9^. SMR is an oscillatory thalamocortical rhythmic pattern of brain activity with a spectral peak frequency of 12–17 Hz measured above the sensorimotor cortex^10^. Modulation of brain circuitries, by increased SMR through neurofeedback brain training, mitigates parkinsonian signs in parkinsonian non-human primates and in PD patients, despite the absence of dopamine-synthesizing cells in the brain^7,9^. SMR activity reduces the sympathetic nerve system via the red nucleus (RN), which is an intermediate between the thalamus and the cerebellum, by controlling the fusiform fibers of the muscle spindles that regulate motor function^11,12^. The cerebellum does not only play a role in the above-mentioned compensation mechanism, but also in the cause of PD by abnormal sensorimotor integration in which the connections with the basal ganglia, cerebellum and thalamus play a key part^13^. Then again, targeting pathways involving a cerebellar circuit might be a promising strategy to prevent susceptibility for PD development and to bypass the dysfunctional basal ganglia in order to restore normal motor function (Fig. 1).

Both the *substantia nigra* and the neighbouring elliptical RN are subcortical centres that play a role in motor coordination^14^. The *substantia nigra* sends excitatory dopaminergic output to the basal ganglia. This basal ganglia plays an important part in the striato-thalamo-cortical pathway for the coordination of motor function (Fig. 1). On the other hand, the two-way connection of the RN with the cerebellum and the cerebral cortex allow its participation in motor control^15^.

Interestingly, post-mortem brain of PD patients shows an increased size of the RN of 32% compared to the size in healthy control brains^16^ suggesting an increased activity of the RN to compensate for the dysfunctional thalamo-striato-cortical circuit.

The RN is composed of two parts, a magnocellular part (RMC) with giant cells and a parvocellular part (RPC) with small cells rostral to the giant cells. In humans and non-human primates, the RMC is relatively small compared to the RPC^17^. However, in other mammals, as rodents and cats, the RN consists mainly of the RMC, rather than the RPC^18^. It seems that being a quadrupedal (walking on 4 paws) animal, is implicated for developing mainly the RMC^19,20^. Also during the crawling infancy stage of human beings, the RMC is involved in the motor coordination until they learn to walk. The use of the RMC is therefore more important in the crawling motion of babies and used at the developmental stage^19^. As soon as the infants learn to walk, a shift towards a predominant role for the RPC takes place. The exact role of the RPC, which is strongly developed in human and monkeys, is not yet known, but it is part of the cerebello-rubro-thalamo-cortical pathway: the inputs from cerebellar nuclei and cerebral cortex project via the RPC to the ipsilateral inferior olive. It is suggested that these connections of the RN with the cerebellum and cerebral sensorimotor cortex allow its participation in motor control^21^.

To examine the role of the RN in compensation mechanisms of PD, we have compared the size of the RN between unaffected and affected parkinsonian monkeys during the presymptomatic stage of MPTP induced PD. Furthermore, we examined the effect of stimulation of a compensation mechanism by SMR brain training on the size of the RN.

## Results

### MPTP treatment procedure

In part 1, in which the susceptibility for developing PD symptoms during the presymptomatic stage was tested, the MPTP dose used was 0.5 mg/kg/week (total dose of 2.5 mg/kg s.c. in the abdominal area; injection-volume of 0.5 ml/kg).

In part 2, in which the compensatory mechanism is activated by SMR neurofeedback, the MPTP dose used was 1 mg/kg/day (excluding the weekend) (total dose of 8 mg/kg s.c. in the abdominal area; injection-volume of 0.5 ml/kg).

#### The relation between the RN size and the susceptibility for developing PD during the early presymptomatic stage

**Figure 2:**
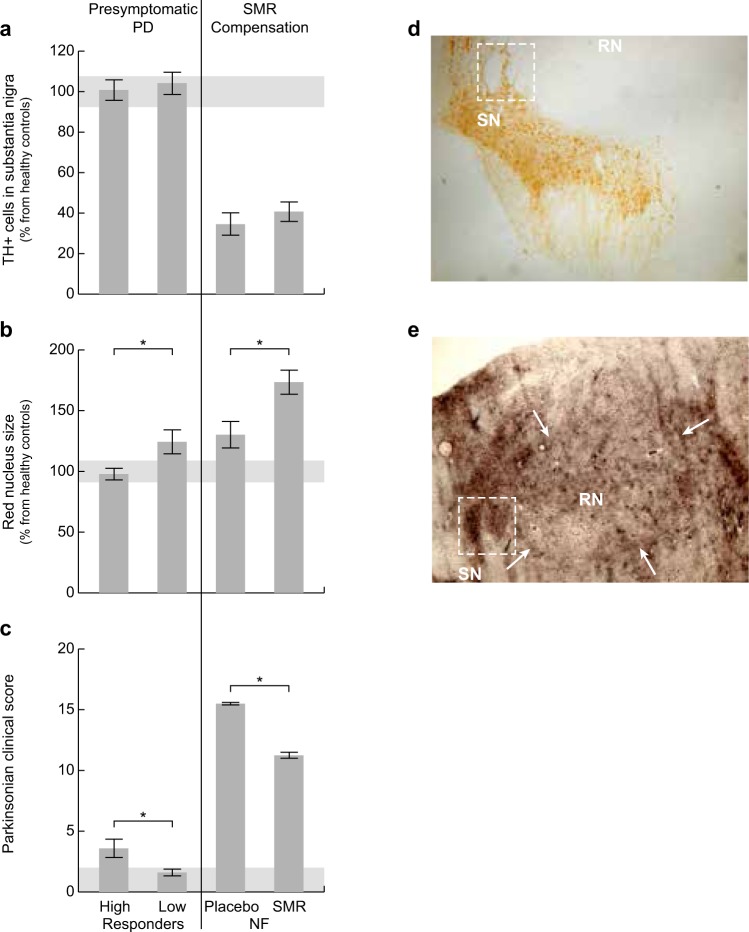
Histological parameters and clinical scores. Histological parameters and clinical scores during the early presymptomatic PD compensation (high vs low responders) and the SMR-induced compensation by neurofeedback (NF) training (placebo vs SMR). *Significantly different between groups (P < 0.05). Horizontal grey areas indicate the value range of healthy controls (n = 7). (**a**) TH-positive cells in the substantia nigra as a percentage of healthy controls (±SEM). During the presymptomatic PD stage no significant differences on cell survival was found between low responders and high responders (n = 6/group, t = 0, d.f. = 9.721, P = 0.6757). During the SMR NF procedure no significant differences was found between placebo trained and SMR trained groups (n = 5/group, t = 0.7361, d.f. = 7.092, P = 0.4853). (**b**) RN size as a percentage of healthy controls (±SEM). The RN was significantly larger in the low responders compared to the high responders (n = 6/group, t = 2.41, d.f. = 7.157, P = 0.046) and in the SMR trained group compared to the placebo trained controls (n = 5/group, t = 2.723, d.f. = 7.831, P = 0.0267). The RN of the placebo trained parkinsonian controls was not significantly increased compared to healthy controls (n = 7, grey bar) (t = 1.944, d.f. = 8.524, P = 0.0856). (**c**) Average (±SEM) clinical score of parkinsonian signs. The high responders show early parkinsonian signs compared to the low responders (F_1,150_ = 108.9, P < 0.0001). The SMR trained monkeys showed a significant reduction of the clinical parkinsonian signs compared to the placebo trained controls (F_1,22_ = 402.5, P < 0.0001). At the right site (**d**,**e**), an example of each staining is given from corresponding brain slices of a high responder monkey (M08112) from experiment 1: (**d**) picture of a TH-IR stained substantia nigra (SN) and (**e**) picture of a Campell-Switzer stained red nucleus (RN). Arrows indicate the border of the RN. The squares (**d**,**e**) indicate the same structure and are used for navigation.

For this experiment twin monkeys from different breeding families were selected. The grouping of the monkeys was based upon the appearance of the clinical signs independent of their familial relationship and resulted in two groups, the so-called ‘high responders’ (n = 6) and ‘low responders’ (n = 6). Clinical scores above 2.0 (high responders) is defined as related to PD. Differences in clinical scores between the high responders and low responders were present already in the second week after the first MPTP injection significant (slope analysis; d.f. = 10, p < 0.001)^22^. In Fig. 2a it is shown that a weekly low MPTP dose of 0.5 mg/kg for five weeks did not induce significant brain damage of tyrosine hydroxylase immune reactivity (TH-IR) positive neurons in the *substantia nigra* compared to healthy controls (P > 0.05). There was also no difference between low responders and high responders (104.2 ± 6.02% vs 100.8 ± 5.07% cell survival, unpaired t-test with Welch’s correction, t = 0, d.f. = 9.721, P = 0.6757, two-tailed).

**Figure 3:**
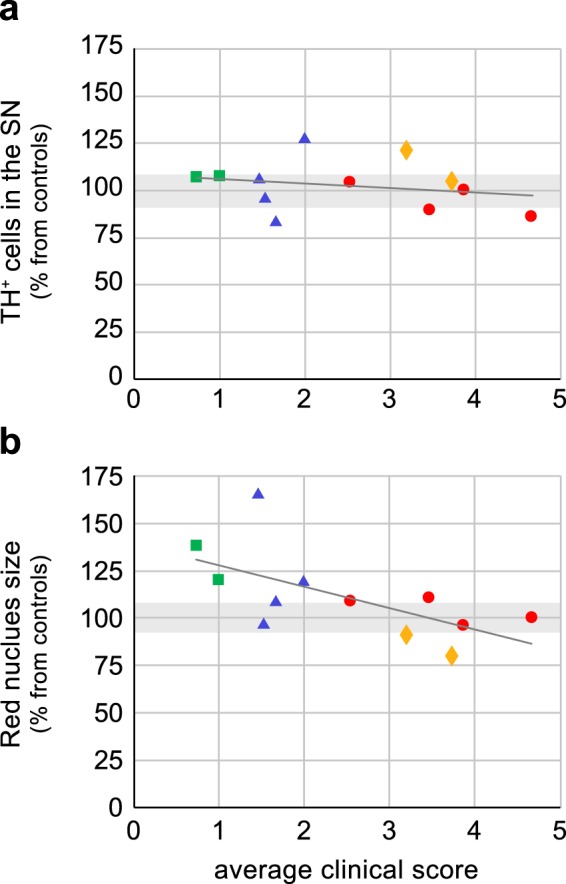
Correlation between histological parameters and clinical scores of parkinsonian signs during the presymptomatic PD compensation. Data of the high and low responders (n = 12) of part 1 are used. (**a**) No correlation was found between the severity of the clinical scores and the number of TH-IR positive cells in the substantia nigra (Pearson r, two-tailed, R^2^ = 0.05603, P = 0.4589). (**b**) A significant correlation was found between the severity of the clinical scores and the size of the RN (Pearson r, two-tailed, R^2^ = 0.3964, P = 0.0283). Individual animals are shown from four different breeding families (square green, triangle blue, circle red and rhombic yellow). Grey areas indicate the range of TH-IR positive cells in controls (a, 100 ± 7.68%), and the range of RN-size in healthy controls (b, 100 ± 9.0%).

Additionally, proteomic markers in the striatum did also not differ between the both groups of low and high responders^22^. Although in the absence of dopaminergic cell loss, the high responder group did show early parkinsonian signs (mean: 3.59 ± 0.76) compared to the low responders (mean: 1.60 ± 0.28) (Two-way ANOVA, F_1,150_ = 108.9, P < 0.0001) (Fig. 2c). Remarkably, monkeys from the same family had a strong tendency to cluster on the expression of clinical signs (Fig. 3).

No correlation was found between the number of TH-IR positive neurons in the *substantia nigra* and the appearance of clinical signs (Pearson r, two-tailed, R^2^ = 0.05603, P = 0.4589) (Fig. 3a). In contrast to the unaffected *substantia nigra*, the relative size of the RN was significantly larger in the low responder group (n = 6) compared to the high responder (n = 6) (124.3 ± 9.93% vs 97.83 ± 4.73%, unpaired t-test with Welch’s correction, t = 2.41, d.f. = 7.157, P = 0.046, two-tailed) (Fig. 2b). A significant correlation was found between the size of the RN and the appearance of clinical signs (Pearson r, two-tailed, R^2^ = 0.3964, P = 0.0283) (Fig. 3b).

#### The relation between the activation of compensatory mechanism by SMR neurofeedback training and the size of the RN in parkinsonian monkeys

For this experiment two groups of monkeys were used. One group was trained for SMR brain activity by neurofeedback before PD induction, the other group was placebo trained in the same test set-up keeping all other factors standardized. In Fig. 2a it is shown that a subacute induction with daily MPTP injections of 1 mg/kg resulted in a >50% cell loss of TH-IR positive neurons in the *substantia nigra* compared to healthy controls (P < 0.01), without difference between SMR neurofeedback (n = 5) and control group (n = 5) (40.75 ± 4.76% vs 34.56 ± 6.93% cell survival, unpaired t-test with Welch’s correction, t = 0.7361, d.f. = 7.092, P = 0.4853, two-tailed).

Although the damage in the *substantia nigra* was equal for both groups, the SMR trained monkeys showed a significant reduction of the clinical parkinsonian signs (11.24 ± 0.25) compared to the placebo trained monkeys (15.49 ± 0.13) (Two-way ANOVA, F_1,22_ = 402.5, P < 0.0001) (Fig. 2c). In contrast to the absence of differences between the TH-IR positive cells in the *substantia nigra*, the relative size of the RN was significantly larger in the SMR trained group (n = 5) compared to the placebo trained parkinsonian control group (n = 5) (173.4 ± 10.36% vs 130.2 ± 12.01%, unpaired t-test with Welch’s correction, t = 2.723, d.f. = 7.831, P = 0.0267, two-tailed) (Fig. 2b). Although the RN of the parkinsonian control group was also increased compared to healthy controls, this difference was not significant (unpaired t-test with Welch’s correction, t = 1.944, d.f. = 8.524, P = 0.0856, two-tailed) (Fig. 2b). The increase of the RN was caused by an increase of the RPC part as there was no increase of the average number the magnocells of the of RMC part in the seven selected slices (control: 17.2 ± 2.4, n = 5 vs SMR: 21.6 ± 2.1, n = 5) (unpaired t-test with Welch’s correction, t = 1.378, d.f. = 7.835, P = 0.2064, two-tailed).

## Discussion

Both, a pre-existing resilience^22^ and the activation of compensatory mechanism^7^ result in reduced expression of clinical parkinsonian symptoms. During the early stage, in which presymptomatic PD compensation is active, it was found that monkeys with a larger RN did not show parkinsonian signs after mild PD induction (Fig. 3). As no differences between low and high responder monkeys were found on markers measured in the *substantia nigra* and striatum, such as cell loss and proteomics^22^, the difference in appearance of early clinical signs is presumably a result of predisposition for compensation for the disturbed striato-thalamo-cortical pathway, in which the RN is involved.

Remarkably, related monkeys from the same families show a comparable size of the RN related to a reduced disease expression^22^. Probably the inherited larger RN makes it easier for the monkeys to compensate during the presymptomatic PD stage.

If the RN indeed plays a role in the compensation mechanism, it might be that activation of neuronal compensatory mechanisms would not only mitigate the parkinsonian signs, but also affect the RN by increased activation. This would explain why the RN in PD patients is larger compared to healthy individuals^16^. This plasticity helps the brain to cope with neurodegenerative conditions in order to maintain regular functioning. In that case the damage triggers the neurological compensation. But does extra stimulation of a neurological compensatory mechanism further improve the condition of the PD subject? Indeed, in a previous study it was shown that stimulation of the SMR brain activity by neurofeedback brain training mitigates the parkinsonian symptoms compared to placebo trained parkinsonian monkeys, whereas the number of TH positive cells in the *substantia nigra* was equally reduced in both groups^7^. This similarity in cell loss indicates that the improvement of the clinical signs was not caused by a neuroprotective effect but rather by a compensatory symptom control effect. Although it was shown that the compensation mechanism is not dopamine mediated^23^, an increasing synergistic effect was found during daily L-DOPA treatment in the SMR neurofeedback-trained monkeys over time^7^. Although placebo effects might play a role in patients, a comparable effect was found in PD patients that underwent SMR neurofeedback training in combination with biofeedback based on respiratory rate^9,24^. A PD patient was able to lower her L-DOPA dose while the PD symptoms were improved. Owing to the damaged basal ganglia in PD there is less innervation of the subthalamic nucleus (STN), which subsequently resulted in a 48% reduction of the STN size in PD compared to healthy control brain^16^. The re-activated STN by L-DOPA administration activates the cerebellar circuits via the pedunculopontine nucleus (PPN) that overrules the disturbed striato-thalamocortical circuit (Fig. 1). Additionally, the hyperdirect pathway, with direct connections between the cortex and the subthalamic nucleus (STN), also acts as a compensatory pathway in PD^4^. This explains that the degree of beta band brain activity in the STN correlates inversely with motor impairment^25^. Therefore, spontaneous exaggeration of slow beta oscillations within the basal ganglia cortical networks seems to be a result of a compensatory mechanism in PD rather than a pathophysiological marker^26^. In healthy human subjects, decreased SMR amplitude was associated with more controlled motor behaviour^27^. This may explain the suppressed signs of uncontrolled movements in PD using the combination of SMR training and L-DOPA treatment.

Remarkably, the histological examination of the brains from the SMR neurofeedback study revealed a significant increase of the RN after stimulation of the SMR compensation mechanism (Fig. 2b) resulting in an improved disease condition (Fig. 2c). Then again, this finding, together with the observation that there is a significant reverse relation between RN size and disease expression during the presymptomatic stage of PD, indicates that the RN plays an important role in motor control and might be involved in the compensation of a damaged striato-thalamo-cortical circuit in human as well as in animal models for PD.

The SMR neurofeedback brain training enhanced compensatory mechanisms that are comparable with the presymptomatic phase of PD^3^ and the paradoxical movement^5^. Both compensatory mechanisms make use of cerebellar circuits that compensate for the impaired thalamo-striato-cortical circuit to maintain the motor function. In these compensation mechanisms as well as during SMR brain activity the RN is involved in the coordination of movements. In primates, especially in humans, the motor control has evolutionary switched from a cerebello-rubro-thalamo-cortical pathway to a more prominent focus on the striato-thalamo-cortical pathway in which the *substantia nigra* plays an important role. This switch went conjointly with a differentiation of the RN showing a strongly reduced RMC-size compared to the RPC in humans and non-human primates^17,28^. In quadrupedal mammals, the RN consists mainly of the RMC part^18–20^. This difference in RN composition can be confusing since the function of the RN has also been transformed. In human both RN parts are completely independent, whereas, in lower mammals the parts are essentially homogeneous^29^. The evolution-derived transition from the rubrospinal to the corticospinal tract towards bipedalism might explain the spontaneous recovery of parkinsonian symptoms in quadruple species who still uses the rubrospinal system for motor control^30,31^. Another important factor in species differences for the expression of parkinsonian symptoms might be the differences in dopamine innervation of the thalamus that is abundant in the primate and only rudimentary in rodents^32^. The striatum in primates became more important and complex in the shift from a rubrospinal system to a corticospinal system during evolution, not only because of differentiation in hand and foot use, but also because of the development of speech and language in human^33^. As the cerebello-rubro-thalamo-cortical pathway is still active in quadruple animals for motor control, it explains why these animals better compensate for the damage in the basal ganglia resulting in a fast recovery of motor symptoms after PD induction^31,34^. Although the exact role of the different parts of the RN remains unknown, its involvement in motor control, like the *substantia nigra*, is evident. Not only through the connections with the sensorimotor cortex, but also strong connections of the RN with the prefrontal cortex are detected by the use of MRI diffusion tensor imaging, suggesting well-organised motor coordination by the human RN^15^.

In conclusion, the fact that a damaged striato-thalamo-cortical pathway and the activation of a compensatory pathway initiate an increase of the RN, combined with the observation of having a larger RN reduces the expression of parkinsonian symptoms, indicate an important role of the RN in alternative pathways in the brain to compensate for striatal damage. The alternative compensation pathways might also explain the spontaneous recovery of parkinsonian symptoms in rodents and cats and provide an opportunity for new targets for symptom control in PD in order to improve the quality of life for PD patients.

## Methods

### Marmoset brain tissue

Brain material from 29 adult common marmoset monkeys (*Callithrix jacchus*), from BPRC’s purpose-bred colony, of both sexes (300–450 gram), 2–4 years of age, of two PD studies were used^7,22^. All monkeys were experimental naïve, pair-housed in spacious cages under intensive veterinary care and controlled conditions compliant with European Community guidelines with a varying enriched cage environment, daily fed with standard monkey-chow (Special Duit Services, Witham, Essex, UK), fruits, vegetables and ad libitum water supply. Both study protocols and experimental procedures were reviewed and approved by the Institute’s Ethics Committee, according to Dutch law (DEC#638 and DEC#660). All the methods were carried out in accordance with the relevant guidelines and regulations.

### Study design

The role of the red nucleus (RN) was examined in two compensatory mechanisms of Parkinson’s disease (PD), (1) presymptomatic PD and (2) sensorimotor rhythm (SMR) activation. The histological data of both studies were compared with a healthy control group.

#### The relation between the RN size and the susceptibility for developing PD during the early presymptomatic stage

Twelve Marmosets (6 M/6 F) were once weekly subcutaneously injected with a very low dose of the neurotoxin MPTP (0.5 mg/kg) for a period of 5 weeks until the first parkinsonian signs appeared (total cumulative MPTP dose: 2.5 mg/kg). The disease progression induced by MPTP was monitored daily by observations of clinical signs and once weekly by motor-related behavioural test. Five days after the last MPTP injection, animals were euthanized under anaesthesia in order to collect brains for histological examination.

#### The relation between the activation of compensatory mechanism by SMR neurofeedback training and the size of the RN in parkinsonian monkeys

Ten marmosets (5 M/5 F) were trained (2–3 times each week) with positive reinforcement on a defined cortical electroencephalogram (EEG) pattern of brain activity above the sensorimotor cortex by neurofeedback technology. Brain activity was recorded using epidural bioelectric bipolar electrodes connected to a bio-potential channel transmitter (Data Sciences International, Transoma medical, Arden Hills, USA) for telemetric registration of the EEG as described earlier^8^. Half of the monkeys (n = 5) were trained on SMR EEG (12–17 Hz). The other five monkeys served as controls that were trained on random EEG in the same test set-up, keeping all other factors standardized. Ten weeks after the start of the SMR neurofeedback training, all marmosets were injected with MPTP (1 mg/kg, s.c.) on consecutive week days (total cumulative MPTP dose: 8 mg/kg). The neurofeedback training continued on weekly basis until the end of the study. The disease progression was monitored by daily observations for clinical signs by a technician unaware of the treatment. Five weeks after PD induction, all monkeys were treated with the anti-PD drug L-DOPA (Madopar, 12.5 mg/kg p.o. BID for 3 weeks). At the end of the study the monkeys were euthanized under anaesthesia in order to collect brains for histological examination.

The histological results of both studies were compared with a control group of naïve, age and gender-matched healthy marmosets (n = 7). These control marmosets were not exposed to any treatment before. Animals were euthanized under anaesthesia in order to collect the brains for histological examination of the *substantia nigra* and the RN.

### Drugs

MPTP (1-methyl-4-phenyl-1,2,3,6-tetrahydropyridine hydrochloride, purchased from Sigma Aldrich, St. Louis, Mo., USA) was dissolved in 0.9% saline to a concentration of 1 mg/ml (for the first part of the study) and 2 mg/ml (as a free base) (for the second part of the study) resulting in a MPTP-dose of respectively 0.5 mg/kg and 1 mg/kg.

L-DOPA (Madopar, 12.5 mg/kg p.o. BID for 3 weeks) was dissolved in Gum Arabic (5 ml/kg). Arabic Gum powder from the Acacia tree (Spray Dried), for oral administration, was purchased from Fagron Ltd, UK. Gum Arabic is a supplementary food source for marmoset monkeys.

### Behavioural observations and measurements

The body weight was measured weekly and on the days of drug administration. The monkeys were daily observed in a blinded manner on parkinsonian symptoms (apathy, inadequate grooming, immobility, muscle rigidity and rest tremors). The clinical symptoms are scored from 0–4 depending on the severity of the symptom expression: 0 (not observed), 1 (observed but normal for the monkey), 2 (mild expression), 3 (moderate expression), and 4 (severe expression). Additional behavioural data are published elsewhere^7,22^.

### Histology

#### Substantia nigra

The *substantia nigra* was analyzed for the presence of dopamine positive neurons with tyrosine hydroxylase immune reactivity (TH-IR) staining as described by Franke *et al*.^22^. Serial brain sections of 7 μm were fixated with acetone for 10 minutes and washed for 5 minutes with PBS 0.1 M (PH 7.4) and Tween20 0.05%. Endogenous peroxidase oxidase was quenched with the DAKO Dual Endogenous Enzyme Block (EnVisiontm G/2 Double Stain System, Rabbit/Mouse (DAB+/Permanent Red, code K5361). Thereafter the sections were pre-coated in PBS with 5% bovine serum albumin (BSA) and 5% Human AB serum for 10 minutes, followed by incubation in anti-Tyrosine Hydroxylase (TH) clone TH-2 (Sigma 1:100), in PBS + 1% BSA + 2% TritonX100 for 10 minutes at room temperature. The secondary anti-body Polymer/HRP (anti rabbit/mouse, DAKO, Glostrup, Denmark) was incubated for 10 minutes. Thereafter, the sections were stained for 10 minutes with 3′3′-diaminobenzidine (DAB+) Chromogen diluted in substrate buffer containing 0.1% hydrogen peroxide to visualize bound immune-complexes. PBS-washes (0.1 M) were applied after each incubation step. After a tap water wash the sections were dehydrated in alcohol series, cleared up in xylol and cover-slipped.

Every 30st section was taken for analysis. Within seven corresponding sections, the total number of TH-IR positive neurons was counted in a blind-manner and expressed as a percentage of the number of cells counted in healthy untreated control marmoset brains.

#### Red nucleus

RN was analysed for its size with the Campell-Switzer staining. Serial sections of 7 μm were fixated with acetone for 10 minutes and washed for 5 minutes with PBS 0.1 M (PH 7.4) and Tween20 0.05%. Afterwards, the slides were transferred to the silver attachment substance, consisting of 1% silver nitrate, 1% potassium carbonate and pyridine for 40 minutes. The sections were then sequentially placed in 1% citric acid and 4.99 pH acetate buffer to stop the attachment of the silver particles. Subsequently, the chemical transformation took place in order to visualize the metallic silver by placing the slides in the physical developer fluid (2–3 minutes), a mixture of sodium carbonate, ammonium nitrate, silver nitrate, tungstosilicic acid and formaldehyde diluted in distilled water. The development was stopped and fixated by sequentially placing the slides in 4.99 pH acetate buffer, distilled water and 0.5% sodium thiosulfate. Afterwards, the slides were dehydrated and mounted with malinol. After a tap water wash the sections were dehydrated in alcohol series, cleared up in xylol and cover-slipped. The size of the RN was detected by indication of three spots of the RN boundary by a technician unaware of the treatment, after which the computer draws an epileptic circle and calculates the area based on the magnification factor. This was repeated in seven systematically sampled sections, with a distance between two sections of approximately 420 μm (every 60th section), throughout the entire RN of each marmoset. Within the seven sections, the total area of the RN was indicated and averaged per treatment group. The average area of the naïve control group was set at 100%. The area of the RN was expressed as a relative value in percentages of the controls.

### Statistics

For the statistical evaluation, between-group comparison is performed using independent t-tests with Welch’s correction. The clinical score data was analysed with linear mixed-effects model fit by residual maximum likelihood estimations (REMLs). A significance level of p < 0.05 was considered significant (Prism 6.0e for Mac OS X; GraphPad Software, San Diego California USA). Justification of the number of animals: The total number of monkeys used was 29 to determine the development of PD signs and symptom reducing effect of SMR neurofeedback (resp. 6 and 5 for each group and additional 7 monkeys for the overall healthy control group). The statistical exact power calculation is based on simple between group t-tests. The formula used is: N = 2(Zα/2 + Zβ)^2^ * (SD/ES)^2^. The calculation is based on an α of 0.05 and a power of 80%. With α set at 0.05, Zα/2 = 1.96; β set at 0.2 (80% power), Zβ = 0.84; 2(Zα/2 + Zβ)^2^ = 15.7. We have selected behavioural observation as primary outcome measure. The SD for parkinsonian behavioural signs based on previous experiments is estimated to be 8. With SD of 8, and effect size 16: N = 15.7 * (8/16)^2^ = 4 (assuming a normal distribution). To adjust to student t distribution the N is increased to 5 (4 + 1 = 5) for each group. As we used twins in the presymptomatic PD part of the project we increased to number of animals to an even number of 6 for these experiments.

## Data Availability

All relevant datasets generated during and/or analysed during the current study are available from the corresponding author on reasonable request. The raw data is stored at BPRC on a paper version of the scoring scale forms. The slices of tissue used for the pathology are stored at BPRC. The results are imported into Excel for further analysis. All originals are present in the archives of BPRC. All data information is stored at BPRC under project number 542394 and 638419.
